# Children’s Healthy Living (CHL) Program for remote underserved minority populations in the Pacific region: rationale and design of a community randomized trial to prevent early childhood obesity

**DOI:** 10.1186/1471-2458-13-944

**Published:** 2013-10-09

**Authors:** Lynne R Wilken, Rachel Novotny, Marie K Fialkowski, Carol J Boushey, Claudio Nigg, Yvette Paulino, Rachael Leon Guerrero, Andrea Bersamin, Don Vargo, Jang Kim, Jonathan Deenik

**Affiliations:** 1 Cancer Research Center of Hawaii, University of Hawaii, (701 Ilalo St #600), Honolulu, HI (96813), USA; 2 Department of Human Nutrition, Food, and Animal Sciences, University of Hawaii at Manoa, (1955 East-West Rd), Honolulu, HI (96822), USA; 3 Office of Public Health Sciences, University of Hawaii at Manoa, (1960 East-West Rd), Honolulu, HI (96822), USA; 4 School of Nursing & Health Sciences, University of Guam, (UOG Station), Mangilao, Guam (96923), USA; 5 Guam Cooperative Extension Service, College of Natural & Applied Sciences, University of Guam, (UOG Station), Mangilao, Guam (96923), USA; 6 Center for Alaska Native Heath Research, University of Alaska Fairbanks, 311 Irving I, Box 757000, Fairbanks, AK(99775), USA; 7 Community and Natural Resources, American Samoa Community College, (PO Box 5319), Pago Pago, American Samoa (96799), USA; 8 Cooperative, Research, Extension and Education Service, Northern Marianas College, (PO Box 501250), Saipan, MP (96950), USA; 9 Department of Tropical Plant and Soil Sciences, University of Hawaii at Manoa, (3190 Maile Way), Honolulu, HI (96822), USA

## Abstract

**Background:**

Although surveillance data are limited in the US Affiliated Pacific, Alaska, and Hawaii, existing data suggest that the prevalence of childhood obesity is similar to or in excess of other minority groups in the contiguous US. Strategies for addressing the childhood obesity epidemic in the region support the use of community-based, environmentally targeted interventions. The Children’s Healthy Living Program is a partnership formed across institutions in the US Affiliated Pacific, Alaska, and Hawaii to design a community randomized environmental intervention trial and a prevalence survey to address childhood obesity in the region through affecting the food and physical activity environment.

**Methods/Design:**

The Children’s Healthy Living Program community randomized trial is an environmental intervention trial in four matched-pair communities in American Samoa, the Commonwealth of the Northern Mariana Islands, Guam, and Hawaii and two matched-pair communities in Alaska. A cross-sectional sample of children (goal n = 180) in each of the intervention trial communities is being assessed for outcomes at baseline and at 24 months (18 months post-intervention). In addition to the collection of the participant-based measures of anthropometry, diet, physical activity, sleep and acanthosis nigricans, community assessments are also being conducted in intervention trial communities. The Freely Associated States of Micronesia (Federated States of Micronesia, and Republics of Marshall Islands and Palau) is only conducting elements of the Children’s Healthy Living Program sampling framework and similar measurements to provide prevalence data. In addition, anthropometry information will be collected for two additional communities in each of the 5 intervention jurisdictions to be included in the prevalence survey. The effectiveness of the environmental intervention trial is being assessed based on the RE-AIM (reach, effectiveness, adoption, implementation, maintenance) framework.

**Discussion:**

The Children’s Healthy Living Program environmental trial is designed to focus on capacity building and to maximize the likelihood of sustainable impact on childhood obesity-related behaviors and outcomes. The multiple measures at the individual, community, and environment levels are designed to maximize the likelihood of detecting change. This approach enhances the likelihood for identifying and promoting the best methods to promote health and well-being of the children in the underserved US Affiliated Pacific Region.

**Trial registration:**

NIH clinical trial # NCT01881373

## Background

### Epidemiology of child obesity

Global prevalence estimates indicate that in 2004 approximately 10% (150-160 million) of school-aged children (5 to 17 years) were overweight, and 2-3% (35-40 million) were obese [1]. In the United States (US), the prevalence of obesity alone was estimated at 17% among 5 to 19 year-olds in 2009-2010 [2]. However, adequate prevalence data are lacking on overweight and obesity in the Pacific Region.

The health and social consequences of excess weight are substantial, and obese and overweight children are at risk for serious chronic illnesses. For example, being overweight and obese is a major risk factor for type 2 diabetes in children [3,4]. Obese children are also more likely than their peers to experience negative social and psychological consequences including discrimination, stigmatization, and low self-esteem [5-8].

Most researchers use body mass index (BMI) to measure child overweight and obesity; however, the BMI levels defining overweight and obesity may differ across countries depending on the reference data and cutpoints used [9]. Global estimates are often based on the World Health Organization cutpoints, which define overweight as > 1SD and obesity as > 2SD above the average (M) where M and SD are derived from a reference population for 5 to 19 year-olds, using data sets compiled by WHO [10,11]. In the US, in children aged 2 to 19 years, overweight is usually defined as greater than or equal to the 85^th^ percentile but less than the 95^th^ percentile and obesity as greater than or equal to the 95^th^ percentile, according to the BMI-for-age Centers for Disease Control and Prevention (CDC) growth charts [12].

### Prevalence of obesity in the US affiliated pacific region

Data from the National Health and Nutrition Examination Survey (NHANES), a program of studies designed to assess the health and nutrition status of adults and children in the US, are used to monitor the trends in child overweight and obesity [13]. The NHANES program, however, does not include data from the noncontiguous US states of Alaska and Hawaii or the US Affiliated Pacific Island Jurisdictions of American Samoa (AS), Commonwealth of the Northern Marianas Islands (CNMI), Guam, the Federated States of Micronesia (FSM), the Republic of Palau, and the Republic of the Marshall Islands (RMI) [14]. According to the few data sources available for the Pacific, the prevalence of child overweight and obesity combined (≥85^th^ percentile BMI-for-age) has been estimated at 22% (of 2 – 5 year olds) in Alaska [15], 32.6% (of 5 – 8 year olds) in Hawaii [16], 33.5% (of 2-10 year olds) in the Commonwealth of the Northern Mariana Islands [16], 33.7% (of 2 – 5 year olds) in American Samoa [17], and 38.5% (of 5-18 year olds) in Guam, using US CDC reference data [18]. Although these estimates are informative, it is important to note that data are derived from small samples and may not be representative of the population.

Overweight and obese combined (≥85^th^ percentile BMI-for-age) prevalence estimates from NHANES in 2007-2008 [19], among 2-5 year olds, are 21.2% overall and 26.0%, 27.7%, and 27.7% for the minority populations of non-Hispanic black, total Hispanic, and Mexican American children, respectively. Among 6-11 year olds from NHANES in 2007-2008 overweight and obese combined (≥85^th^ percentile BMI-for-age) prevalence estimates are 35.5% overall and 37.6%, 42.6%, and 41.7% for the minority populations of non-Hispanic black, total Hispanic, and Mexican American children, respectively. The 2009-2010 cycle of NHANES documents similarly high prevalence among some US minority groups (Black, Mexican American) [2]. These estimates are similar to the existing estimates from the noncontiguous US states and US Affiliated Pacific Island Jurisdictions. Data from the Supplemental Nutrition Program for Women, Infants and Children (WIC) in 2006 estimate prevalence of combined overweight and obesity (≥85^th^ percentile BMI-for-age) in children ages 2-5 years at 34.7% for Native American/Alaska Native and 33.4% for Native Hawaiian/Pacific Islanders [20]. In contrast, the estimates among the Asian, Black, and White children are 17.9%, 20.8%, and 21.5%, respectively [20].

Despite the serious underrepresentation of the Pacific Region in obesity research, the high prevalence of chronic conditions in the region has prompted the US Affiliated Pacific Islands (AS, CNMI, Guam, FSM, Palau and RMI) to declare a “State of Emergency” [21]. Evidenced by the high proportions of Pacific Island adults who are overweight and obese, prevention of overweight and obesity among children in the Pacific as primary prevention of chronic conditions is of great focus and concern [22].

### Establishment of CHL

The Children’s Healthy Living Program for Remote Underserved Minority Populations in the Pacific Region (CHL) is a partnership among the remote Pacific jurisdictions of Alaska, AS, CNMI, the Freely Associated States of Micronesia (FAS including RMI, Republic of Palau, FSM), Guam, and Hawaii to study child obesity among Pacific children, ages 2-8 years. The program is sponsored by the United States Department of Agriculture (USDA), Agriculture and Food Research Initiative. To address the child obesity epidemic in the Pacific, the CHL partners have identified the following program objectives: 1) Conduct program/data inventories and situational analysis; 2) Train 22 professionals and paraprofessionals in obesity prevention; 3) Develop a Pacific food, nutrition, and physical activity data management and evaluation system; 4) Develop and conduct a community-based environmental intervention to prevent, maintain, or reduce young child overweight and obesity; 5) Evaluate the environmental intervention; and 6) Incur at least one obesity prevention policy change per jurisdiction.

**Figure 1:**
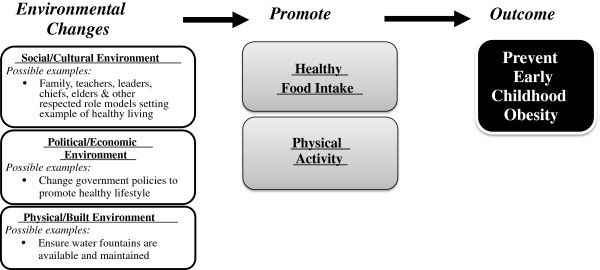
The Children’s Healthy Living Program model to influence multiple aspects of the environment to promote healthy food intake and physical Activity in young children (2 - 8 years) as a method to prevent early childhood obesity in the US Affiliated Pacific.

The focus of this paper is on objectives 4-6, which relate specifically to the rationale and design of a community randomized environmental intervention trial in five CHL jurisdictions, and the evaluation of these efforts. Intervention planning involves the modification of three aspects of the environment (social/cultural, physical/built, political/economic) to improve the diet quality and physical activity level of young children (See Figure 1). The methodology and results of program/data inventories and situation analysis, critical to the design of the strategy and content of the community intervention, has been published elsewhere [23].

The investigators hypothesized that children in communities receiving the CHL obesity prevention intervention would differ from their counterparts in communities not receiving the CHL obesity prevention intervention. All successful components of the CHL intervention are planned to be implemented in the comparison communities after the trial is completed (i.e., delayed “optimized” intervention). Specifically, the investigators hypothesized that, relative to children in the comparison communities, children in the intervention group will have the following differences:

1) lower weight and BMI velocities.

2) increased sleep.

3) reduced consumption of sugar-sweetened beverages (SSB).

4) higher fruit and vegetable intake.

5) higher water intake.

6) reduced TV/video viewing.

7) increased physical activity.

8) lower prevalence of acanthosis nigricans (AN).

The CHL intervention targeted the following changes: a reduction in 0.08 of BMI z-score, an addition of 15 min/day of sleep, a reduction of 0.5 cups/day of SSB, an increase of 1 serving/day of fruits and vegetables, an increase of 0.5 cups/day of water, a reduction of 10 min/day of TV/video viewing, and a reduction of 5% in AN. The targets are goals of the intervention and provide guidance for messaging. The differences that the CHL study is powered to detect are provided in the Power and sample size calculations section below.

Previous randomized controlled trial studies in young children, 2-8 years of age, that focused on at least one of CHL’s primary outcomes, showed a positive effect of intervention on body mass index (BMI), food intake, and/or physical activity (PA) [24-29]. Multiple intervention strategies were used in another community trial that were shown to be safe and effective in reducing unhealthy weight gain in children, without increasing health inequalities [30]. That study included efforts to modify children’s food intake and physical activity, and parental involvement with the children around food and physical activity. However, data on PA expenditure and on overall diet quality of participants in these trials are limited. In addition, the populations included in most of these studies are whites in the US, Australia, or Europe. There is little known on the efficacy of such actions in remote diverse populations of Pacific Islander, Native Hawaiian or Alaska Native ethnicity. CHL will be able to fill these gaps in the peer reviewed literature.

## Methods/Design

The CHL Community Randomized Trial is an environmental intervention trial in five jurisdictions: Alaska, AS, CNMI, Guam, and Hawaii. The Freely Associated States of Micronesia, which includes FSM, the Republic of Palau, and RMI, will conduct elements of the CHL sampling framework and measurements to provide prevalence data, but are not engaged in the community intervention trial. The CHL Program was designed to monitor and evaluate the prevalence of obesity in the region through the baseline and follow-up data, while testing the intervention for impact by comparing intervention with non-intervention communities.

The community intervention activities are quantitatively and qualitatively evaluated [31] in Alaska, AS, CNMI, Guam, and Hawaii to determine the impact of the intervention. The intervention was developed through blending findings from a community engagement process that allowed the community to identify and prioritize strategies [23] and review of evidence based literature.

Institutional Review Board approvals from the University of Alaska Fairbanks, University of Guam, and University of Hawai'i at Manoa were attained prior to data collection. The American Samoa Community College and the Northern Marianas College, and the institutional partners in the Freely Associated States of Micronesia ceded their Institutional Review to the University of Hawai'i at Manoa.

In addition, approvals for working with teachers and parents of Head Start (a US federally funded program that educates preschool-age children and their families) and government-sponsored Early Childhood Education programs in the Freely Associated States of Micronesia were received in coordination with the program directors and/or boards, when appropriate. Other local level agreements included approvals from the chiefs (matai) and ministers (faifeau) of participating American Samoan villages and the mayors in participating villages in Guam.

### Site selection process

Communities were identified in Alaska, American Samoa, CNMI, Guam and Hawaii using the 2000 US Census tract data since 2010 data was not available at the census tract level [32] at the time that sites were selected in 2011. In the FAS, 2010 country census data were used to inform selection of sites for prevalence survey data collection [33-35]. Community selection was based on the following eligibility criteria: population size of >1000, >25% of the population of indigenous/native descent (15% in Alaska due to no census tract with a population of more than 1000 having more than 25% indigenous/native), and >10% of the population under age 10 years (based on combining census tract data groups of < 5 years of age and 5 – 9 years of age, in order to have sufficient population size for CHL target of 2 to 8 year olds). Additional selection criteria included having adequate settings for sampling children (e.g., schools); evidence that children live and go to school in the same community (i.e., not a commuter community), ensuring that the measured children have an opportunity to be exposed to the intervention; a minimal risk of contamination between matched-pair communities; having reasonable accessibility for the CHL team (e.g., isolated communities that would require substantial travel logistics were excluded); community cohesiveness [36]; having sufficient settings for intervention (e.g., community centers, parks, churches, and stores); and for the FAS, scheduled air or boat service. A list of all eligible communities was created in each of the jurisdictions based on the above criteria.

**Figure 2:**
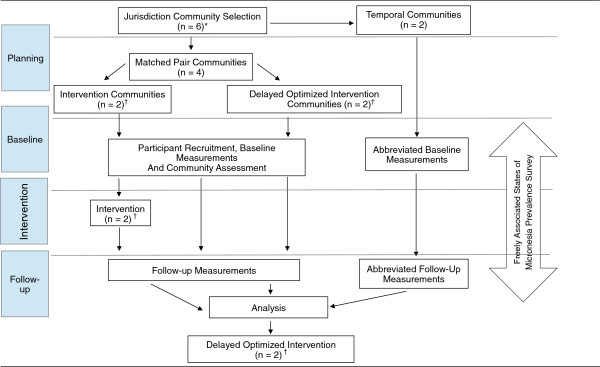
**Children’s Healthy Living Program study design schematic.** *Alaska will only include 4 communities. ^†^Alaska will only include 1 community.

Communities in each of the five jurisdictions were selected to participate in the intervention trial. In American Samoa, CNMI, Guam and Hawaii, four communities were selected, while two communities were selected in Alaska due to large distances between sites. The communities were matched to form pairs (1 matched-pair in Alaska and 2 matched-pairs for the other jurisdictions; see Figure 2). The pairs were formed by matching communities on the following factors: percentage in poverty based on U.S. census, population density based on U.S. census, distance from urban centers, and percentage overweight/obesity, when available. In each pair, one community was randomly assigned to intervention and the other to a delayed optimized intervention (community will receive intervention at the end of the main trial).

Randomization to intervention, in general, produces study groups that are comparable with respect to confounding variables [37]. An independent statistician performed the randomization.

Two additional non-matched communities (third and fourth for Alaska and fifth and sixth for other jurisdictions) were selected from the eligible list to serve as temporal indicators of anthropometry status (see Figure 2). Generally, the communities selected for temporal assessment had been considered to participate as a matched pair; however, they often did not match another eligible community well or they had less community cohesiveness, which was not as important for a community providing prevalence information only. The temporal communities will not receive the intervention program,

In the FAS region, 3-5 communities were selected for collection of baseline (prevalence) survey data in each of Chuuk, Kosrae, Pohnpei, Yap, Palau and the RMI (n=200 children per location), according to the same criteria, plus a criteria of geographic representation. A total of 27 communities will provide baseline (prevalence) survey data from the FAS.

Thus, in total, four communities in Alaska and six communities in each of the remaining four CHL intervention jurisdictions were selected for a total of twenty-eight communities across the CHL region for participation in the CHL community intervention trial: 9 matched pairs (18 sites total) and 10 temporal sites.

A cross-sectional sample of children in each of the CHL intervention communities is being assessed for outcomes at baseline and at 24 months (18 months post-intervention). Additionally, the outcomes are being assessed in the FAS region to provide prevalence information.

The intervention does not explicitly target the assessed children; they serve as representatives of their communities. Children who participate at both time points provide repeated measures and serve as an embedded longitudinal sample.

### Power and sample size calculations

Sample size estimates were based on the need for a sufficient number of communities and children in each of the five jurisdictions to ensure adequate statistical power to detect meaningful differences between intervention arms in overweight and related outcomes (listed previously) overall and for select outcomes within jurisdictions. The effect size (Cohen’s d) [38] was calculated based on an analysis of 2000 simulated data sets with children clustered within community clustered within jurisdiction. The intervention effect was tested based on an F test of the interaction term of intervention group and time from a mixed model of the outcomes, accounting for the clustering in a group-randomized trial (GRT) by adjusting the test degrees of freedom to the number of communities [39]. The calculations assume a minimum n of 150 children with anthropometry and a minimum n of 100 children with accelerometry and food and activity logs in 6 communities in four jurisdictions and in two communities in Alaska; this assumption is conservative as the goal is a sample size of 180 children per community. An expected correlation for communities within jurisdictions was low with an estimate of the interclass correlation coefficient (ICC) that varied between 0.02 to 0.04. We assumed a critical level of 0.05 (two-sided), a power of 80%, and a constant sample size at baseline and at 24 months. The respective effect sizes for an ICC of 0.02 and 0.04 are modest at 0.26 and 0.35 for outcomes with n=150. Using means and variances for the outcomes from previous research [24,40,41], the minimum detectable differences for the two ICC values were 0.09 and 0.12 for BMI z-score, 21 and 28 minutes of television viewing, and 11 and 15 minutes of sleep. The respective effect sizes for an ICC of 0.02 and 0.04 are also modest at 0.31 and 0.42 for outcomes with n=100. Using means and variances for the outcomes from previous research [24,40,42,43], the minimum detectable differences for the two ICC values were 0.50 and 0.67 servings of vegetables, 0.45 and 0.61 servings of fruits, 0.45 and 0.60 servings of water, 0.34 and 0.46 servings of SSB, and 33 and 45 minutes of PA with metabolic equivalent values (METs) > 3, based on accelerometry.

### Participant recruitment goals

In order to meet sampling goals for children between the ages of 2 – 8 years, recruitment activities involve schools and other community venues and activities. Recruitment sites consist of Head Starts, pre-schools/day cares, kindergartens, WIC sites, community health centers and other appropriate venues (e.g., parks and community recreation centers). Recruitment efforts, led by CHL staff in each jurisdiction, involve close collaboration with community liaisons (e.g., teachers, school staff, program directors, matai, mayors) to enhance participation and retention throughout the measurement protocol. The teams in all jurisdictions tailored the recruitment strategies to work effectively with the stakeholder organizations while meeting recruitment goals of CHL. The total proposed sample size for anthropometry measures for CHL is 4100 children for the cross-sectional samples at baseline and at 24 months. For the embedded longitudinal (individual) design, the intent is to collect repeated measurements from 40-50% of children with baseline measurements. To ensure an adequate representation of 3-5 year olds at baseline for the embedded longitudinal sample, 70% of the sample was targeted from Head Starts, pre-schools or day cares and 30% of the sample was targeted from kindergartens (or pre-schools with a sufficient number of 5 year olds).

### Study design

**Figure 3:**
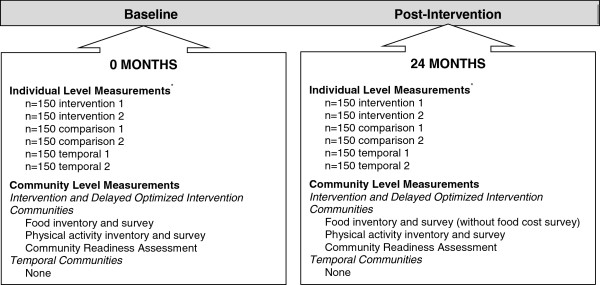
**The Children’s Healthy Living (CHL) Program individual and community level measurement timeline.** *Longitudinal sample will be embedded in the cross-sectional sample.

The CHL Program is as an 18-month community randomized environmental intervention trial focused on preventing early childhood obesity and promoting a healthy diet and physical activity in young children in the Pacific Region (Novotny R PI, USDA Grant award 2011-68001-30335, 4/1/2011 – 3/31/2016). The trial is registered with NIH (Novotny, clinical trial # NCT01881373). Baseline measurements began in October 2012. The measurements focus on both participant (child) measures and community assessments, and are being repeated post-intervention (October 2015; see Figure 3). The intervention began in January 2013 in intervention communities when baseline measurements were complete. Measurements in the FAS region, focusing on establishing prevalence data for the region, are targeted for completion in September 2014.

All matched-pair communities are assessed during a 12-month baseline measurement period and at post-intervention using the same protocol and procedures. Community assessments of each matched-pair community are conducted within the same time frame as the child measures. In each matched-pair, intervention communities were prioritized for measurement so that the intervention study phase could commence in January 2013. Participant and community assessment measurements are listed in Table 1.

**Table 1:** The Children’s Healthy Living (CHL) Program individual and community level measures

**Individual level measures**				**Assessed in matched-pair communities**		**Assessed in temporal communities**		**Assessed in FAS**^**†**^
**Category**	**Measurement**	**Measurement tools**	**Completed by**	**0 month**	**24 month**	**0 month**	**24 month**	
Demographic	Demographic[15,43-48]	Questionnaire	Surrogate*	X	X	X	X	X
Anthropometry	Height	Stadiometer	Staff	X	X	X	X	X
	Weight	Portable Scale	Staff	X	X	X	X	X
	Waist circumference	Circumference Tape	Staff	X	X	X	X	X
Diet	2 d^#^ Food intake[61,62]	Food & Activity Log	Surrogate*	X	X			X
Physical Activity (PA)	6 d PA[66]	Accelerometer**	Child	X	X			X
	2 d^#^ Activity Log [62]	Food & Activity Log	Surrogate*	X	X			X
Sedentary behavior (SB)/Screen Time (ST)	6 d SB/ST[66]	Accelerometer**	Child	X	X			X
	2 d^#^ Activity Log[62]	Food & Activity Log	Surrogate*	X	X			X
	Usual SB/ST[52]	Questionnaire	Surrogate*	X	X			X
Sleep	6 d Sleeping[66]	Accelerometer**	Child	X	X			X
	2 d^#^ Activity Log[62]	Food & Activity Log	Surrogate*	X	X			X
	Sleeping behavior[53]	Questionnaire	Surrogate*	X	X			X
Acanthosis Nigricans	Presence/Severity[67]	Visual observation/ assessment form	Staff	X	X			X
Culture	Language/culture[49-51]	Questionnaire	Surrogate*	X	X			X
**Community level measures**				**Assessed in matched-pair communities**		**Assessed in temporal communities**		**Assessed in FAS**^†^
**Category**	**Measurement**		**Completed by**	**0 month**	**24 month**	**0 month**	**24 month**	
Food[69-71]	Food Outlet Inventory		Staff	X	X			X
	Fast Food Restaurant Inventory		Staff	X	X			X
	Fast Food Observation Form		Staff	X	X			X
	CHL Food Cost Survey		Staff	X				X
	Food Availability & Marketing Survey		Staff	X	X			X
	Store Environment Walkability Survey		Staff	X	X			X
Physical Activity[69,74]	Physical Activity Facility Inventory		Staff	X	X			X
	Physical Activity Facility Observation Form		Staff	X	X			X
	Park Inventory		Staff	X	X			X
	Park Observation Form		Staff	X	X			X
	School Inventory		Staff	X	X			X
	School Observation Form		Staff	X	X			X
	Inventory for Church and Community Centers Used for Physical Activity		Staff	X	X			X
	Church Observation Form		Staff	X	X			X
Community Medical Facilities[76,77]	Medical Facility Inventory Form		Staff	X	X			X
Community Readiness[78]	Community Readiness Assessment		Key community Stakeholders	X	X			

^†^FAS = Freely Associates States of Micronesia.

X = indicates measurement completed.

*Surrogate reporter = parent/caregiver.

**A minimum of 100 children in each matched-pair community and FAS jurisdiction will wear an accelerometer.

^#^Randomly assigned non-consecutive days.

Measurements are collected in either a school setting (e.g., Head Start), or in a community-based setting (e.g., Community Recreation Center) and require a minimum of two visits. At the first visit, study participants’ parents/caregivers are oriented to the study, provide informed consent and complete questionnaires. Participants receive instructions on completing a food and activity log, and on the use of an accelerometer (Actical, Murrysville, PA). If children are present (e.g., at a community-based setting), accelerometers are placed, and anthropometric measures and acanthosis nigricans screening are performed, after receiving assent from the child. At the second visit, 6 days later, the accelerometers are removed and the food and activity logs are collected and reviewed with the parent/caregiver. Any children not measured in visit one for anthropometry and acanthosis nigricans are measured at the follow-up visit. Compensation for study participation was provided at visits 1 and 2 ($10 each in Hawaii, American Samoa and CNMI and increased by investigators in Guam to $20 and in Alaska to $25 based on their determination of a locally acceptable level). A similar procedure is followed in the FAS prevalence study, where $10 is provided at each of visits 1 and 2.

Abbreviated measures are completed in temporal communities during transition periods between matched-pair communities or after all matched-pair community assessments are completed. The scheduled visits are limited to one visit, which consists of an orientation to the study, informed consent, the completion of a demographic questionnaire, and the collection of the child’s anthropometric measurements. Measurement settings included both school settings and community-based settings. Compensation for participation was provided at the end of visit 1 ($7 in each of Hawaii, American Samoa, and CNMI, $10 in Guam and $25 in Alaska). Community assessments are not conducted in the temporal communities. Additionally, recruitment group leaders (teachers, community leaders) are provided with $20 for their assistance with recruitment groups in all jurisdictions (including the FAS).

### Individual level outcomes

Anthropometric changes are the primary health outcomes of the study, and anthropometry is measured in all communities. Diet, physical activity, and sedentary behavior are additional primary behavioral outcomes measured in matched-pair and FAS communities. Sleep and acanthosis nigricans are secondary outcomes measured in the matched-pair and FAS communities. All field research staff underwent measurement training and standardization of anthropometric measurements, and a thorough review and testing of protocols and procedures were conducted, before baseline measurement collection was initiated.

#### Participant and family/household characteristics and behaviors

At the first visit, in matched-pair and FAS communities, parents/caregivers complete four questionnaires. The first questionnaire assesses the demographic profile of the child including age, race/ethnicity, sex, household composition, educational level and income of the parent/caregiver, household food security, religion, general health status, and early life feeding behaviors. Questions have been adapted from those previously used in other studies [16,44-49]. The second questionnaire relates to the cultural identity of the parent/caregiver. This questionnaire was developed for Native Hawaiians and has been adapted [50-52] to be generalizable to the entire CHL region. The third and fourth questionnaires on usual screen time/sedentary behavior and sleep, respectively, have been adapted from previous studies [53,54], with terminology added to the usual screen time/sedentary behavior [53] and sleep [54] questionnaires for clarity. The forms are administered in English in Alaska, CNMI, Guam, and Hawaii. In other jurisdictions, the forms are translated into other languages. The translated versions of the forms were back-translated as the standard protocol [55] to ensure the correct content has been conveyed in the translation. In American Samoa, the forms are translated into Samoan, and in the FAS region, into Chuukese, Kosraen, Marshallese, Onouan, Palauan, Pohnpein, Ulithian and Yapese.

#### Anthropometry and body composition

Weight, height, and waist circumference are measured by trained research staff based on standardized procedures and protocols [56-58]. Zerfas criteria are used to standardize research staff against the height, weight, and waist measurement of a certified anthropometrist (Novotny R, CHL PI) [59]. Zerfas provides no waist circumference criterion; however, the uniform criterion assigned to all assessments measured in cm (mm) units is used. No research staff can assess children for a measure for which they did not pass the Zerfas criteria.

Participants wear lightweight clothing and no shoes, and empty their pockets. Height is measured to the nearest 0.1 cm using portable stadiometers (Perspective Enterprises, PE-AIM-101; Portage MI). Weight is measured to the nearest 0.1 kg using portable scales (Seca Model 876; Chino CA). Plastic tape (Seca Model 201; Chino CA) is used to measure waist circumference at the level of the umbilicus to the nearest 0.1 cm [56]. Weight, height, and waist circumference measurements are measured three times; three additional measures are made if there are no two measures among the original three within 2 units (e.g., 0.2 kg for weight). These measures are used to compute Body Mass Index (BMI) as weight (kg) / height (m)^2^, waist (cm) to height (cm) ratios, and subsequently BMI z-score, waist circumference z-score, BMI-for-age-percentiles, and waist circumference-for-age percentiles [60,61].

#### Dietary intake of children

Food logs (i.e., dietary records), reported by a surrogate (parent or other caregiver), are used to assess energy, nutrient, and food group intake of the child. The format and methods used for the food logs have been adapted from previous studies [62,63]. The food log is combined with an activity log in an easy-to-carry booklet, referred to as the Food and Activity Log (FAL). Parents/caregivers are asked to complete the FAL for their children on two randomly assigned non-consecutive days, which include weekdays and weekend days, between visit 1 and 2. Assignment of recording days is based on the day of the child’s first visit (Monday – Saturday). Standard techniques are used to improve accuracy of information recorded in the FAL [64]. Parents/caregivers are instructed in record keeping techniques with the aid of food models, service ware, and utensils. Parents are provided a tool kit of calibrated utensils (i.e., measuring cups and spoons); the FAL; and a Ziploc® (Racine WI) bag in which to place food wrappers, labels, and packages (WLP). CHL staff follow-up with reminder telephone calls. During visit two, research staff review the FAL with the parents (e.g., for completeness of food entries, portion size estimation, food preparation methods, accuracy of recording data). Staff are trained to enter the FAL data into the Pacific Tracker3 (PacTrac3) dietary and physical activity assessment program [63]. Data from the PacTrac3 are used to calculate food groups and nutrients using a food composition database developed by the University of Hawaii Cancer Center for use in the Pacific region [63,65,66].

#### Child activity

The Actical accelerometer (Z series, Phillips Respironics Inc; Murrysville PA) is a small, lightweight, water resistant, omni-directional device capable of measuring movement in multiple planes and providing data on intensity, frequency, and duration of activities in young children [67]. Devices are initialized to save data in 1-second intervals to record spontaneous movements of young children. The device is worn on the participant’s non-dominant wrist attached with an Ident-A-Band (tear-resistant; Hollister; San Fernando CA) plastic band. Participants are asked to wear the device daily (without removal) until it is removed by research staff 6 days later. The participants and their parents/caregivers are assured that the devices could be worn while bathing, swimming or sleeping. Extra bands are provided for situations where the device comes off, is removed or has to be replaced. At the conclusion of data collection, the accelerometer data are processed using the manufacturer’s software (Actical version 3.0) with output activity in counts/minute.

Research staff instruct parents/caregivers to record their child’s activities for the same two days in which food intake is recorded on the FAL. Parents are instructed to provide the start and end time and details of each activity, including sleep and screen time, throughout a 24-hour period. Activity data are entered using PacTrac3, which calculates minutes of activity by intensity level and METs.

#### Acanthosis nigricans (AN)

Participants’ necks are examined for the presence of AN by two trained research staff. Using Burke’s quantitative scale for AN, a score is given for AN severity: 0 to 4 [68]. Participants with a score of one or higher are considered to have AN. AN is independently associated with hyperinsulinemia, an important risk factor for type 2 diabetes [69], so parents/caregivers of participants with a positive screen for AN are provided with a referral to follow-up with their children’s health care providers or a public health service provider.

### Community level outcomes

#### Community assessment toolkit (CAT)

The CHL CAT has been created to evaluate the food and physical activity environment of each matched-pair and FAS community. The evaluation of the food environment includes an inventory and survey of fast food restaurants and food outlets adapted from surveys from the CX3 and BTG (Bridging the Gap) programs (see Table 1) [70-73]. A food cost survey, adapted from the Alaska Food Cost Survey [74], is also being conducted. The evaluation of the physical activity environment includes an inventory and survey of physical activity facilities, parks, schools, and churches and community centers used for physical activity, also adapted from surveys from the BTG program (see Table 1) [70,75]. A walkability checklist has been adapted from a U.S. Department of Transportation Federal Highway Administration and National Highway Traffic Survey Administration survey [76]. An inventory of medical facilities in the region is also included, adapted from a survey from the CHANGE (CDC Community Health Assessment and Group Evaluation) program [77]. Staff conduct initial information gathering for inventories using on-line resources followed by in-person visits to complete the inventories and to perform the surveys. The information from the CAT is used to inform the design of the intervention and is used to aid in the interpretation of results by relating changes in behavior to changes in the environment.

#### Community readiness assessment (CRA)

The CRA has been modified from the Community Readiness Structured Interview Tool used in the Obesity Prevention in Communities (OPIC) Study [78], designed to be implemented via a paper-based or electronically delivered (e.g., web link) survey. Key informants who are knowledgeable about the food and activity environment of the target communities are identified and invited to complete the CRA pre- and post-intervention. To gather an accurate account of community readiness, a minimum of five respondents per community is required. At post-intervention, key informants are identified through the same process with an effort made to interview the same key informants, if possible. The CRA assessed 6 dimensions of community readiness: Efforts, Knowledge of Efforts, Leadership, Attitude, Knowledge about the Issue, and Resources for Prevention Efforts. Responses are averaged per community to produce a Community Readiness Score, a quantitative estimate of community capacity [78]. Scores range on a scale from 1 (No Awareness) to 9 (High Level of Community Ownership) [78]. The community readiness score is used to develop appropriate and specific nutrition and physical activity interventions for each community and is used as an adjustment factor in the evaluation of intervention effectiveness.

#### Intervention effectiveness evaluation

The effectiveness of the CHL intervention is assessed based on the RE-AIM framework. The RE-AIM framework has been developed to enhance the impact of health interventions through evaluating integral components for sustainable change (e.g., will the CHL intervention have the capacity to reach the underserved populations of the Pacific and lead to the adoption of healthier eating and activity behaviors) [31,79]. This approach to evaluating intervention effectiveness has successfully been used in a physical activity intervention in children from Hawaii [80]. Reach and adoption is determined through monthly process intervention evaluations from each jurisdiction. Effectiveness of the CHL intervention is determined by evaluating the outcome data (change in BMI, servings of water, minutes of physical activity, etc.). Implementation is determined through intervention mid-point implementation quality evaluations. Post-intervention (~6 months), the number of CHL-influenced activities still ongoing is evaluated to determine maintenance of the intervention. This approach to applying RE-AIM is similar to what has been done in other studies [81].

## Discussion

The purpose of this paper is to present the rationale and the design of the CHL community based intervention trial. CHL’s study design is a multi-component, field-based, community-engaged research trial that integrates measurement with the intervention components. Baseline environmental assessments are incorporated into the baseline measurements. Process evaluation during the intervention provides evidence of the quality of intervention and feedback into improving and re-targeting the intervention delivery for delayed optimized communities. Process evaluation ensures that the intervention is related to the desired outcomes of the CHL Program. The CHL intervention trial examines effectiveness and sustainability using the RE-AIM framework [31,79], which addresses organizational level adoption, implementation, and program maintenance, encouraging the sustainability of positive intervention components.

One of the innovations of CHL is the focus on the immediate environment where children live, eat, and play. There are multiple intervention touch points throughout each community providing various avenues to reach the members and children of the community. The CHL trial is designed to focus on capacity building and to maximize the likelihood of sustainable impact on childhood obesity-related behaviors and outcomes. The multiple measures at the individual, community, and environment levels are designed to maximize the likelihood of detecting change. This is appropriate for such an undertaking to ensure learning and to promote the health of the children in the underserved Pacific Region.

## Abbreviations

AN: Acanthosis nigricans; AS: American samoa; BMI: Body mass index; BTG: Bridging the gap program; CAT: Community assessment toolkit; CDC: Centers for disease control and prevention; CHANGE: CDC Community health assessment and group evaluation program; CHL: Children’s healthy living program; CNMI: Commonwealth of the Northern Mariana Islands; CRA: Community readiness assessment; FAL: Food and activity log; FAS: Freely Associated States of Micronesia (includes RMI, Republic of Palau, FSM); FSM: Federated States of Micronesia; GRT: Group-randomized trial; ICC: Interclass correlation coefficient; METs: METabolic equivalent values; NHANES: National health and nutrition examination survey; NIH: National institutes of health; OPIC: Obesity prevention in communities study; PA: Physical activity; RE-AIM: Reach, effectiveness, adoption, implementation, maintenance; RMI: Republic of the Marshall Islands; SSB: Sugar-sweetened beverages; USDA: United States department of agriculture; WHO: World health organization; WIC: Special supplemental nutrition program for women, infants and children; WLP: Food wrappers, labels, and packages.

## Competing interests

The authors declare that they have no competing interests.

## Authors’ contributions

All authors participated in conceptualizing the design and coordination of the study. LRW, RN, MKF, CJB, YP, CN, RG, AB, JD lead the drafting and critical revising of the manuscript. All authors read and approved the final manuscript.

## Pre-publication history

The pre-publication history for this paper can be accessed here:

http://www.biomedcentral.com/1471-2458/13/944/prepub
